# Exploring Attachment-Related Factors and Psychopathic Traits: A Systematic Review Focused on Women

**DOI:** 10.3390/bs15091293

**Published:** 2025-09-22

**Authors:** Marina Leonor Pinheiro, Ana Beatriz Machado, Rui Abrunhosa Gonçalves, Sónia Caridade, Olga Cunha

**Affiliations:** 1 School of Psychology, University of Minho, Campus de Gualtar, 4710-057 Braga, Portugal; pg50170@alunos.uminho.pt (A.B.M.); rabrunhosa@psi.uminho.pt (R.A.G.); scaridade@psi.uminho.pt (S.C.); olga.cunha@psi.uminho.pt (O.C.); 2 Psychology Research Center (CIPsi), University of Minho, 4710-057 Braga, Portugal

**Keywords:** attachment-related factors, psychopathic traits, women

## Abstract

Psychopathy in women remains understudied, particularly regarding the role of early relational experiences such as attachment. This systematic review aims to synthesize the current evidence on the association between attachment-related factors and psychopathic traits in women. A structured search following PRISMA guidelines across multidisciplinary databases (Scopus-Elsevier^®^, ESBCO^®^, Pubmed^®^, Sage Publishing^®^, B-On, and Web of Science-Core Collection^®^) yielded 147 articles, of which eight met the inclusion criteria. The majority of the studies met three to four out of the five methodological quality criteria. Findings indicate that insecure attachment styles are differentially associated with psychopathy. Avoidant attachment is positively linked to callous-unemotional traits, whereas the role of anxious attachment appears more heterogeneous. Some studies associate it with higher levels of affective traits and secondary psychopathy, while others identify it as a negative predictor of callous-unemotional features in women. Avoidant attachment was also negatively correlated with empathy and positively related to maladaptive emotion regulation strategies, particularly among women with secondary psychopathic traits. Negative maternal parenting was associated with elevated psychopathic traits, whereas positive parental involvement acted as a protective factor. Additional predictors included early maladaptive schemas and childhood risk factors such as parental criminality and poor supervision. These findings highlight the relevance of early intervention and attachment-informed approaches in forensic risk assessment, trauma-informed care, and prevention programs targeting women with psychopathic traits.

## 1. Introduction

Psychopathy is a complex and heterogeneous personality disorder characterized by deficits in affective processing, interpersonal functioning, and behaviour (Hare, 2003; Guay et al., 2018). Although extensively studied in men, psychopathy in women remains comparatively understudied, despite growing evidence suggesting that it may present distinct developmental trajectories, behavioural manifestations, and clinical implications (Forouzan & Cooke, 2005; Verona & Vitale, 2006). This gender bias in the literature has contributed to gaps in risk assessment protocols and intervention strategies, which are often based on male-oriented models.

Understanding psychopathy in women is crucial for improving the accuracy of forensic assessments, predicting criminal behaviour, and designing gender-sensitive interventions. Attachment theory (Bowlby, 1969) offers a useful framework for exploring how early relational experiences may contribute to developing psychopathic traits. Insecure attachment styles, defined by Bowlby as patterns of relating to others that develop when a child’s early caregiving experiences are inconsistent, unavailable, or rejecting (Bowlby, 1969), have been linked to emotional dysregulation, empathy deficits, and antisocial tendencies (Fraley, 2002)—all of which are characteristic features of psychopathy.

This systematic review aims to synthesize existing evidence on the association between attachment-related factors and psychopathic traits in women. By synthesizing findings from multidisciplinary research, this review seeks to clarify the role of attachment and related factors in the developmental pathways of psychopathy and to underscore their implications for clinical and forensic practice.

## 2. Psychopathy in Women: An Overview

Psychopathy is a severe personality disorder characterized by a combination of interpersonal characteristics such as grandiosity, superficial charm, and deceitfulness; affective traits such as callousness, lack of remorse, and shallow affect; and behavioural tendencies such as impulsivity and irresponsibility (Hare, 2003). This disorder is frequently associated with antisocial tendencies, including early criminality and violations of social norms (Hare, 2003; Hare & Neumann, 2008). Based on the Hare Psychopathy Checklist (PCL-R), often considered the “gold standard” for assessing psychopathy (e.g., Seara-Cardoso et al., 2019), the construct is currently conceptualized through a four-facet model comprising interpersonal, affective, lifestyle, and antisocial components (Hare, 2003; Hare & Neumann, 2008).

Additionally, Karpman (1941) distinguished between primary and secondary psychopathy. Primary psychopathy is typically characterized by low anxiety, emotional detachment, and manipulativeness, while secondary psychopathy is marked by impulsivity, emotional dysregulation, and heightened reactivity, often linked to trauma or early adversity (Levenson et al., 1995). Primary psychopathy is more commonly observed in men, whereas secondary psychopathy is more frequently reported in women (Falkenbach et al., 2017; Moffett et al., 2020). In women, secondary psychopathy often manifests as interpersonal instability, emotional lability, and self-destructive behaviour, which may complicate both diagnosis and treatment planning.

Although psychopathy is less prevalent in women than in men (Forouzan & Cooke, 2005), research indicates that psychopathic traits in women are strongly associated with both violent and non-violent offenses (Eisenbarth et al., 2008) and are linked to increased risk of recidivism, particularly among those with elevated scores on the antisocial component (Pinheiro et al., 2022). However, comparative studies suggest that women with psychopathic traits generally exhibit lower recidivism rates than men (Salekin et al., 1998). Regardless of gender, individuals with high scores on the antisocial and lifestyle components of psychopathy tend to engage more frequently in criminal behaviour than those whose profiles are predominantly interpersonal or affective (Hicks et al., 2010).

The expression of psychopathy also appears to differ between genders. Women with psychopathic traits tend to display greater emotional instability, self-harming tendencies, and relational aggression, whereas men typically exhibit more overt violence and instrumental aggression (Forouzan & Cooke, 2005). Studies focusing on forensic and clinical samples of women with psychopathic traits have shown deficits in recognizing negative emotions such as fear, sadness, anger, and disgust (Dawel et al., 2012; Eisenbarth et al., 2008). Neuroimaging evidence suggests that these emotional impairments are associated with cortical dysfunctions similar to those found in male forensic populations (Eisenbarth et al., 2008). Supporting these findings, lexical decision tasks have shown emotional processing deficits in both men (R. J. Blair, 2007) and women (Vitale et al., 2007) with psychopathic traits. Furthermore, reduced startle modulation—a physiological indicator of blunted emotional reactivity—has been observed in female forensic samples (Eisenbarth et al., 2008), consistent with findings in male populations (Patrick, 1994). These emotional deficits are most strongly associated with the interpersonal-affective traits of psychopathy (Factor 1) in both women and men (Patrick, 1994; Pinheiro et al., 2023). Research also suggests that women with psychopathic traits experience particular difficulty identifying negative emotional states, especially fear and sadness (Dawel et al., 2012), as well as anger and disgust (Eisenbarth et al., 2008). Some evidence indicates that gender may moderate emotional recognition abilities in individuals with psychopathic traits (Delk et al., 2017).

## 3. Attachment: A Perspective of Theory from Childhood to Adulthood

Attachment theory, originally proposed by Bowlby (1969, 1988), offers a foundational framework for understanding how early relational experiences shape emotional development, interpersonal functioning, and long-term personality organization, and in some cases, contribute to maladaptive traits, such as those observed in psychopathy (Christian et al., 2017). While some studies suggest that early attachment patterns may influence relational functioning in adulthood, particularly in intimate relationships (Fraley, 2002; Reis et al., 2000), the notion of stable continuity from childhood to adult attachment remains controversial.

Recent empirical and theoretical contributions have questioned the assumption that early attachment styles remain fixed across development (Raby et al., 2015). Although Bowlby recognized the potential for both stability and change, early attachment research tended to emphasize the stability of internal working models, possibly leading to a publication bias that favoured positive results and underreported null findings (Duschinsky, 2020). A meta-analysis by Verhage et al. (2016) found a decline in effect sizes over time, suggesting that early studies may have overestimated attachment stability. Moreover, longitudinal studies have produced mixed results. While some report moderate correspondence between infant and adult attachment classifications (e.g., Hamilton, 2000; Waters et al., 2000), others show minimal or no continuity (Lewis et al., 2000). These inconsistencies are compounded by conceptual and methodological challenges: the developmental and adult attachment research traditions often use distinct conceptualizations and instruments, limiting comparability and complicating the assessment of continuity (Raby et al., 2015). Together, these findings suggest that attachment should be understood as a dynamic and malleable construct, shaped by ongoing relational experiences and life events—such as parental loss, divorce, abuse, or caregiver mental illness—which may prompt meaningful changes in attachment representations across the lifespan (Duschinsky, 2020; Waters et al., 2000).

This dynamic view of attachment underscores the importance of early caregiving experiences in shaping later emotional and relational functioning. Secure attachment emerges from consistent and responsive caregiving and is associated with effective emotional regulation, trust in others, and the capacity to form stable and supportive relationships (Ainsworth et al., 1978; Fonagy et al., 2002; Mikulincer & Shaver, 2007). However, children who experience maltreatment, separation, or abuse (Schimmenti et al., 2014) tend to develop insecure attachment patterns, marked by avoidance and anxiety (Reiser et al., 2019), which have been increasingly examined in studies about psychopathic traits in adulthood. Insecure attachment in adults has been associated with violence against intimate partners (Noonan & Pilkington, 2020), internalizing and externalizing behaviour problems (Madigan et al., 2016), and vulnerability to psychopathology (Russell & King, 2016). Anxious attachment, which develops from inconsistent caregiving, is characterized by emotional hyperactivation, heightened sensitivity to rejection, and relational dependency (Cassidy & Shaver, 2016; Shaver & Mikulincer, 2002). Avoidant attachment stems from emotionally unavailable or rejecting caregiving and leads to the deactivation of attachment needs, excessive self-reliance, and discomfort with intimacy (Ainsworth et al., 1978; Dozier et al., 2008). Finally, disorganized attachment often arises in contexts of maltreatment, abuse, or trauma, where the caregiver is simultaneously a source of comfort and fear—resulting in contradictory behavioural strategies, affective instability, and heightened vulnerability to psychopathology (Lyons-Ruth & Jacobvitz, 2016; Schimmenti et al., 2014).

## 4. Attachment, Psychopathy, and the Need for Gender-Sensitive Approaches

Given the developmental plasticity of attachment, research on its link with psychopathy in adulthood should be interpreted with caution. Adult attachment styles may not reflect a direct continuation of early-life patterns, but rather the cumulative influence of relational experiences across the lifespan (Thompson et al., 2022). The relationship between attachment and psychopathy is well established across genders, with insecure attachment—characterized by avoidance and anxiety (Schimmenti et al., 2014; Reiser et al., 2019)—being linked to core psychopathic traits such as emotional callousness, affective detachment, and disregard for social norms (Patrick, 2010).

Specific developmental risk factors, such as paternal absence, socioeconomic disadvantage, parental rejection, and emotional neglect, may intensify insecure attachment and inhibit the development of empathy, thereby fostering affective deficits commonly observed in individuals with psychopathic traits (Gaik et al., 2010; Gao et al., 2010).

Empirical research consistently shows that insecure attachment is associated with impairments in emotional regulation and reactivity (Marganska et al., 2013). In particular, avoidant attachment has been linked to antisocial behaviour and externalizing problems (Gaik et al., 2010). Individuals with psychopathic traits often display relational styles marked by low empathy, emotional detachment, and a diminished concern for others—patterns typically consistent with avoidant attachment (Christian et al., 2017). These interpersonal tendencies resonate with the core features of primary psychopathy, characterised by emotional coldness and a lack of interpersonal engagement (Mikulincer & Shaver, 2012; Mooney et al., 2019). By contrast, anxious attachment has been associated with increased emotional reactivity, affective instability, and negative self-perceptions (H. G. Hong et al., 2016). These features closely reflect the emotional dysregulation characteristic of secondary psychopathy (Moffett et al., 2020).

Both men and women with psychopathic traits often exhibit similar attachment deficits and associated affective dysregulation, suggesting some shared underlying mechanisms (Hare & Neumann, 2008). However, despite these similarities, there is compelling evidence for meaningful gender differences in the developmental pathways and manifestation of psychopathy, which underscores the need for gender-sensitive approaches, particularly tailored for women (Verona & Vitale, 2018). From an evolutionary perspective, gender differences in attachment styles provide insight into the development of psychopathic traits. Men typically exhibit avoidant attachment behaviours, consistent with reproductive strategies focused on mating with multiple partners while minimizing parental investment and emotional commitment (Del Giudice, 2009). In contrast, women more commonly display anxious attachment, aimed at securing sustained partner investment and maintaining relationships despite potential personal costs (Del Giudice, 2009). Empirical evidence supports these patterns, with women scoring higher on anxiety attachment, especially during early adulthood (Chopik et al., 2013). While earlier studies reported inconsistent gender differences in attachment (e.g., Collins & Read, 1990), some research confirms these distinctions (Chopik et al., 2013). Women with psychopathic traits tend to present with a distinct clinical profile characterized more frequently by histories of interpersonal trauma, affective instability, and disorganized attachment patterns (Forouzan & Cooke, 2005; Verona & Vitale, 2018). Moreover, women appear more sensitive to relational disruptions and are more likely to engage in emotion-focused coping strategies following attachment-related adversity (Mikulincer & Shaver, 2007).

At the neurobiological level, early attachment disruptions have been linked to alterations in brain development, including impaired white matter integrity in the corpus callosum—an area involved in emotional processing and interhemispheric communication (Kobak & Madsen, 2008; Raine et al., 2003). These structural differences may underlie some of the emotional and interpersonal dysfunctions observed in individuals with psychopathic traits, particularly those resulting from early adversity. In addition to relational factors, genetic predispositions also contribute significantly to psychopathy. Specific genetic variants have been associated with an increased risk of violent and psychopathic behaviours (Tiihonen et al., 2020), and heritable traits such as aggression, emotional callousness, and low empathy appear to be part of the psychopathy spectrum (Beaver et al., 2011). The interaction between genetic vulnerabilities and environmental factors—such as insecure attachment—seems to be reciprocal. For instance, children exhibiting high levels of callous-unemotional (CU) traits may elicit harsher or more inconsistent parenting, which in turn exacerbates their socioemotional impairments (Hawes et al., 2011). Moreover, children with genetic sensitivity to environmental influences may be particularly vulnerable to disruptions in attachment, which further impairs emotion regulation and social adaptation.

Although these differential associations between attachment styles and psychopathy subtypes are theoretically compelling, findings in this area remain inconsistent. This variability reinforces the need for further empirical investigation that accounts for developmental timing, gender, and contextual influences.

## 5. Attachment-Related Factors and Psychopathy: Maternal and Paternal Influences

Although attachment theory provides a fundamental framework for understanding personality development and traits such as psychopathy, it is essential to recognize that attachment-related factors—such as parental sensitivity, emotional communication, and disciplinary practices—also play a crucial role in shaping developmental outcomes (Gaik et al., 2010; Thompson et al., 2022). These variables can mediate or moderate the effects of secure attachment on core outcomes such as emotion regulation, neurobiological maturation, and interpersonal functioning (Thompson et al., 2022). Furthermore, the relationship between attachment and these factors is dynamic and reciprocal, illustrating the plasticity of development, in which child characteristics and caregiver responses continually influence one another over time (Thompson et al., 2022).

Parental influences extend beyond secure attachment and encompass contextual risk factors such as inconsistent discipline, parental rejection, emotional neglect, and adverse family environments, all of which have been associated with elevated psychopathic traits in offspring (Del Gaizo & Falkenbach, 2008; Farrington, 2007). For example, individuals with pronounced interpersonal-affective traits frequently report histories of parental antipathy and neglect (Farrington, 2007). Primary psychopathic traits have been linked to feelings of abandonment and mistrust, whereas secondary traits are more commonly associated with anxious attachment and emotional dysregulation (H. G. Hong et al., 2016).

Although maternal relationships have traditionally received greater attention in this area of research (Bisby et al., 2017), growing evidence underscores the significant and often underexplored influence of paternal factors. Low paternal protection, emotional unavailability, or physical absence have been associated with emotional detachment in later relationships and with increased psychopathy scores in adulthood (Gao et al., 2010). Despite societal changes in parental roles—where fathers increasingly share caregiving responsibilities—the literature continues to focus predominantly on mothers, leaving paternal contributions relatively neglected (Brown et al., 2012). Additionally, structural factors such as low socioeconomic status, family violence, single parenting, or parental psychopathology can further exacerbate risk, suggesting that psychopathic traits emerge from a constellation of interpersonal and environmental adversities rather than attachment alone (Chinchilla & Kosson, 2016).

## 6. Current Study

Understanding the relationship between attachment and related factors and psychopathy has important practical implications, particularly in assessing public safety risks associated with violent or antisocial behaviour. Because psychopathic traits can contribute to interpersonal harm and, in some cases, violence, identifying key developmental risk factors, especially among women, who remain underrepresented in psychopathy research, is essential.

Although there is growing awareness of gender differences in the expression of psychopathy, little research has focused on how attachment-related factors relate to psychopathic traits in adult women. This is especially relevant given that attachment behaviours—and the emotional consequences of insecure attachment—can differ significantly between men and women (Mikulincer & Shaver, 2007). For instance, women may be more sensitive to relational disruptions and respond differently to early experiences of rejection or neglect, potentially influencing the development and expression of psychopathic traits in distinct ways.

This systematic review is guided by the following research questions: (a) To what extent does avoidant attachment predict psychopathic traits in women; (b) how is avoidant attachment associated with callous-unemotional (CU) traits and affective/interpersonal features in women; (c) to what extent is anxious attachment linked to secondary psychopathy and maladaptive emotion regulation; (d) and how do maternal versus paternal influences differently contribute to variations in CU traits and other psychopathy dimensions?

The aim of this review was to synthesize current knowledge on the association between attachment styles and related factors, such as paternal influences, and psychopathic traits in women. To our knowledge, no previous review has specifically addressed this relationship. In doing so, we seek to fill a gap in the literature and offer a gender-informed understanding of how early relational experiences may contribute to the development of psychopathic traits in women.

Importantly, this review emphasizes the need to view psychopathy through a gendered lens to improve risk assessment, guide targeted interventions, and strengthen prevention strategies. By focusing on women, we aim to explore how attachment styles and related factors may uniquely influence emotional development and behavioural outcomes in this population. Because attachment patterns and their psychological effects may manifest differently across genders, this review provides insight into the gender-specific pathways that may underlie the development of psychopathy, and how this knowledge can support more effective responses in clinical, social, and criminal justice contexts.

## 7. Methods

This systematic review followed the Preferred Reporting Items for Systematic Reviews and Meta-Analyses (PRISMA) guidelines (Page et al., 2021). It adopted the PICOS framework (Population, Intervention, Comparison, Outcomes, and Study design) to guide the eligibility criteria and search strategy (Amir-Behghadami & Janati, 2020). The use of the PICOS model ensured a focused and structured approach, allowing for the clear identification of relevant components, specifically: (P) adult women; (I) attachment styles and other related factors (assessed in either childhood or adulthood); (C) not applicable, given the observational nature of included studies; (O) presence or development of psychopathic traits in adulthood; and (S) empirical, peer-reviewed studies of any methodological design.

## 8. Eligibility Criteria

Studies were included if they met the following criteria: (1) participants were women whose attachment or related factors were assessed either during childhood or adulthood, with psychopathic traits assessed in adulthood; (2) participants were from either community or incarcerated samples; (3) studies investigated the relationship between attachment and psychopathic traits or examined parenting variables explicitly linked to attachment theory; (4) studies were empirical, peer-reviewed articles published in English, Portuguese, or Spanish, as these languages matched the authors’ proficiency and ensured accurate interpretation of the data; (5) studies with mixed-gender samples were included only if data for women were analysed separately. The rationale for including studies assessing attachment in childhood lies in the theoretical and empirical evidence suggesting that early attachment patterns tend to persist into adulthood and may significantly shape later socio-emotional and interpersonal functioning, including the development of psychopathic traits (Fraley, 2002; Mikulincer & Shaver, 2007). Although continuity between childhood and adult attachment is debated, with some longitudinal findings showing inconsistencies (Raby et al., 2015; Verhage et al., 2016), a modest degree of stability—especially in emotionally significant relationships—has been observed (Fraley, 2002). Thus, even as attachment is understood as a dynamic construct, examining early attachment remains pertinent for identifying developmental pathways associated with maladaptive traits.

Studies were excluded if they were reviews, theoretical papers, grey literature (e.g., theses, dissertations, conference abstracts), or if they did not clearly measure both attachment or related factors and psychopathy constructs in accordance with established theoretical models.

## 9. Search Strategies

A comprehensive search of six electronic databases—Scopus (Elsevier^®^), EBSCOhost^®^, PubMed^®^, SAGE Journals^®^, B-On, and Web of Science Core Collection^®^—was conducted to identify relevant articles published up to February 2024. The search strategy was designed to be broad and inclusive, reflecting the interdisciplinary nature of research on attachment and related factors and psychopathy.

The search terms were developed based on the central constructs of the review and included both controlled vocabulary and free-text terms. A combination of Boolean operators (AND, OR) was used to combine search terms. The complete search string was: (women OR female) AND (psychopathy OR “psychopathic traits” OR “PCL-R”) AND (attachment OR “attachment styles” OR “adult attachment” OR “child attachment” OR “binding” OR “abusive parenting” OR “insecure attachment” OR rejection OR abandonment OR “avoidance attachment” OR negligence OR “anxious attachment” OR “secure attachment” OR “disorganized attachment”).

The inclusion of terms such as “rejection,” “abandonment,” “abusive parenting,” and “binding” was grounded in their established relevance within the attachment literature, particularly in relation to early relational trauma and the development of disorganized or insecure attachment patterns, which are theorized to influence the emergence of psychopathic traits (Lyons-Ruth & Jacobvitz, 2016).

In addition to the electronic database searches, a manual search was undertaken to ensure the comprehensiveness of the review and to identify any potentially relevant studies not captured by the electronic search. This was conducted after the initial database search and involved reviewing the reference lists of all included studies and key review articles on attachment and psychopathy in female populations.

The review protocol, detailing the search strategy and inclusion criteria, was preregistered on the Open Science Framework (OSF) platform [blind for review], ensuring transparency and adherence to predefined methods.

## 10. Study Selection

All references were imported into Rayyan software (https://www.rayyan.ai) for systematic screening and the removal of duplicates. Titles and abstracts were independently reviewed by two researchers to assess whether the studies met the inclusion criteria. Full texts were then reviewed for all studies that appeared eligible or were unclear based on the abstract. Discrepancies were resolved through discussion with a third researcher. Inter-rater reliability was calculated during the full text screening phase, achieving an agreement rate of 89%, with disagreements resolved by consensus. Reasons for exclusion were documented and are detailed in the PRISMA flowchart.

## 11. Data Extraction

Relevant data from each study were extracted into a codebook, including author(s), year, country, sample characteristics (e.g., size, age, and setting), study design, measures of attachment and psychopathy, main findings, and reported limitations. These data are presented in Table 1 which provides a comparative overview of the studies’ characteristics and results. A narrative synthesis was used to integrate findings due to the heterogeneity in sample types, attachment measures, and psychopathy assessments, which precluded a meta-analytic approach.

## 12. Methodological Quality Analysis

The methodological quality of the included studies was assessed using the Mixed Methods Appraisal Tool (MMAT) (Q. Hong et al., 2018). This tool evaluates studies according to five core criteria appropriate to their methodological design (qualitative, quantitative, or mixed methods). The five MMAT criteria include: (1) clarity of the research questions; (2) adequacy of data collection methods; (3) appropriateness of sampling strategy; (4) quality of data analysis; and (5) consideration of limitations and biases. Each criterion is rated as “Yes”, “No”, or “Can’t tell”, enabling a quality score ranging from 1 to 5. Both reviewers independently assessed all included studies using the MMAT criteria. In cases where quality ratings differed, discussions were held to reach a consensus.

## 13. Results

The findings are delineated across two distinct tables. Table 2 outlines the characteristics of the studies incorporated in this review, whereas Table 1 encompasses the main outcomes of these studies.

## 14. Included Studies

**Figure 1:**
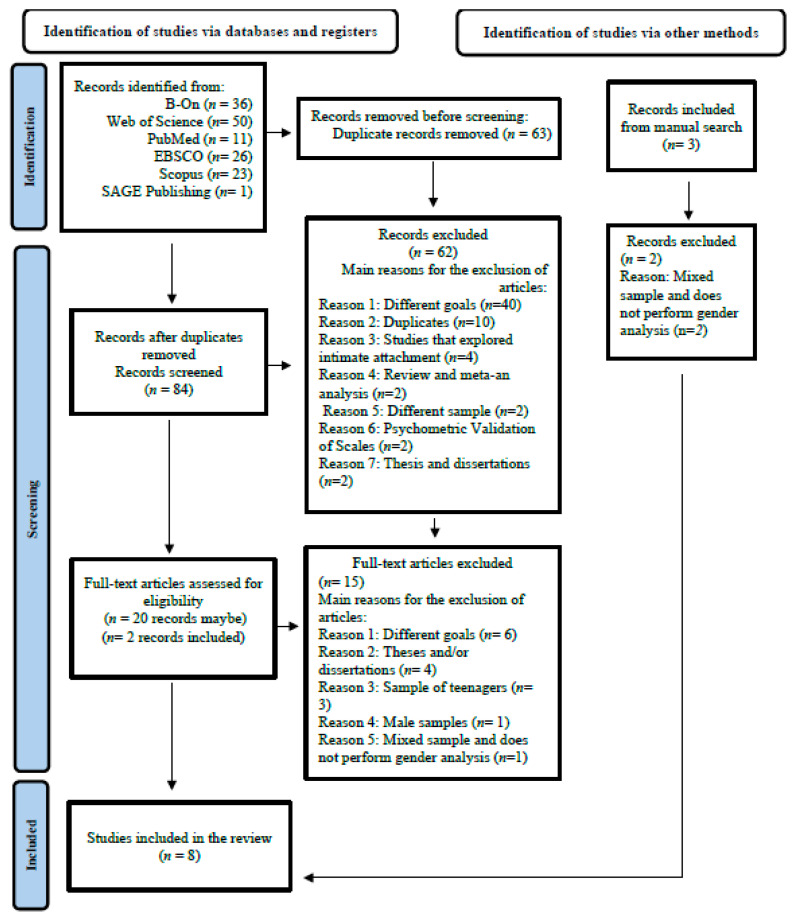
Preferred Reporting Items for Systematic Reviews and Meta-Analyses—Flow diagram of the study selection process. Note. Adapted from Moher et al. (2009).

A total of 147 articles were initially identified across the selected databases. Following the removal of 63 duplicate articles, two independent investigators screened the abstracts and titles of the remaining 84 articles. Among these, 62 articles were excluded for not meeting the defined eligibility criteria, primarily because they explored objectives different from those of the present study (*n* = 40) or were additional duplicates (*n* = 10) (see Figure 1).

Subsequently, the full texts of the remaining articles were thoroughly read, resulting in the inclusion of seven articles in the current systematic review. Additionally, the reviewers conducted a hand-search, identifying three additional articles. However, two of these articles were excluded due to their failure to meet the defined criteria (e.g., Schimmenti et al., 2014), with only one being included (Kyranides et al., 2023). Thus, this systematic review included eight articles that explore the relationship between attachment and related factors and psychopathy in women.

## 15. Quality Assessment

In this systematic review (see Table 3), most studies (*n* = 5) met four out of five quality criteria of the MMAT checklist (Arrigo & Griffin, 2004; Bloxsom et al., 2021; Farrington & Bergstrøm, 2023; Kyranides & Neofytou, 2021; Walsh et al., 2019) and the remaining studies (*n* = 3) met three out of five criteria, indicating a moderate methodological quality (Doroszczyk & Talarowska, 2023; Eisenbarth et al., 2018; Kyranides et al., 2023).

## 16. Characteristics of Studies Included

### Sample Characteristics

Most of the studies (*n* = 7) included in this systematic review were conducted with community samples (Arrigo & Griffin, 2004; Bloxsom et al., 2021; Doroszczyk & Talarowska, 2023; Farrington & Bergstrøm, 2023; Kyranides & Neofytou, 2021; Kyranides et al., 2023; Walsh et al., 2019), while only one study was carried out in a prison setting (Eisenbarth et al., 2018). Half of the studies were conducted in the United Kingdom (*n* = 4; Bloxsom et al., 2021; Farrington & Bergstrøm, 2023; Kyranides & Neofytou, 2021; Kyranides et al., 2023) and three were conducted in the United States of America (*n* = 3; Arrigo & Griffin, 2004; Eisenbarth et al., 2018; Walsh et al., 2019). One study did not specify the country where it was conducted (Doroszczyk & Talarowska, 2023).

Most of the studies included in this systematic review (*n* = 5) were conducted with mixed samples (men and women) and were included because they conducted gender-specific analyses (Doroszczyk & Talarowska, 2023; Farrington & Bergstrøm, 2023; Kyranides & Neofytou, 2021; Kyranides et al., 2023; Walsh et al., 2019). Participant ages ranged from 18 (Bloxsom et al., 2021) to 72 years (Kyranides et al., 2023). The sample size of the qualitative study was one woman (Arrigo & Griffin, 2004), and quantitative studies ranged from 129 (Doroszczyk & Talarowska, 2023) to 752 women (Kyranides et al., 2023).

Most of the studies were non-randomized quantitative (*n* = 6), one was a mixed-methods descriptive study (*n* = 1), and one was a case study (*n* = 1) (see Table 2).

## 17. Main Outcomes

The findings from this systematic review are organized around shared datasets and consistent themes for clarity. The data were organized into two key themes: (1) Attachment Styles and Psychopathy, and (2) Early Life Experiences, Attachment-related Factors, and Psychopathy.

### 17.1. Attachment Styles and Psychopathy

The findings revealed significant positive correlations between both anxious and avoidant parental attachment styles and psychopathy (Bloxsom et al., 2021). Specifically, avoidant attachment emerged as a strong predictor of callous-unemotional (CU) traits in psychopathy, whereas anxious attachment was a negative predictor of CU traits in women (Kyranides et al., 2023). The affective factor of psychopathy showed strong associations with avoidant attachment, while the antisocial factor was negatively associated with anxious attachment (Walsh et al., 2019). Interestingly, secondary psychopathic traits were positively correlated with both avoidant and anxious attachment, although no such correlation was found for primary psychopathic traits (Kyranides & Neofytou, 2021).

Furthermore, Machiavellianism, a key trait in the Dark Triad, was positively associated with both anxious and avoidant adult attachment styles, especially in close relationships, such as those with best friends or intimate partners, suggesting that these attachment tendencies influence adult intimate relationships (Bloxsom et al., 2021).

Doroszczyk and Talarowska (2023) documented positive correlations between psychopathy severity and maladaptive cognitive schemas, including emotional deprivation, mistrust/abuse, entitlement/grandiosity, insufficient self-control, and approval-seeking behaviours. Of these, entitlement/grandiosity and impaired limit-setting domains emerged as predominant predictors (Doroszczyk & Talarowska, 2023). These cognitive-affective structures likely represent early disruptions in secure attachment formation, contributing to emotional detachment and interpersonal dysfunction characteristic of psychopathy.

The included studies also demonstrated that empathy deficits and maladaptive emotional regulation strategies mediate the relationship between insecure attachment and psychopathy in women. In terms of empathy, avoidant attachment was negatively correlated with both affective and cognitive empathy, while anxious attachment showed a positive correlation with affective empathy, particularly in relationships with close friends (Bloxsom et al., 2021). Regarding emotional regulation, secondary psychopathic traits were positively associated with maladaptive strategies such as rumination, self-criticism, and catastrophizing, while being negatively associated with adaptive strategies. In contrast, primary psychopathic traits were positively associated with catastrophizing and, unexpectedly, with adaptive strategies like positive reappraisal (Kyranides & Neofytou, 2021).

When considering only the studies that directly focused on attachment styles and psychopathy (Bloxsom et al., 2021; Kyranides & Neofytou, 2021; Kyranides et al., 2023; Walsh et al., 2019), the overall findings were consistent across studies. Avoidant attachment emerged as a robust predictor of CU traits, whereas the role of anxious attachment was more heterogeneous, showing both risk and protective associations depending on the psychopathy dimension assessed.

### 17.2. Early Life Experiences, Attachment-Related Factors, and Psychopathy

This review identified significant associations between early life experiences, attachment styles, and psychopathic traits in women. These experiences encompass broader contextual and environmental factors, including family criminality, disrupted caregiving, and socioemotional adversity.

Studies revealed that negative caregiving practices were influential factors in exacerbating psychopathic traits among women (Bloxsom et al., 2021; Eisenbarth et al., 2018), while positive parental involvement functions as a robust protective mechanism against the development or escalation of psychopathy (Arrigo & Griffin, 2004). A comparative examination by Eisenbarth et al. (2018) demonstrated that, while both maternal and paternal negative practices correlate with elevated psychopathy scores, maternal dynamics characterized by abuse exhibit stronger predictive validity for psychopathic traits, particularly regarding the psychopathy lifestyle factor. Conversely, paternal influence operates through more indirect pathways, mediated by personality trait configurations (Eisenbarth et al., 2018).

Additionally, maternal and paternal factors that predict CU traits were examined. It was found that mothers’ attachment dimensions, characterized by high levels of a mother’s high regard (e.g., respect for the maternal figure; maternal warmth), negatively predicted CU traits, but this effect was observed only in men. In contrast, a high responsibility (e.g., feeling responsible for the parent) exhibited by fathers was a positive predictor of CU traits, with the effect being observed only in women (Kyranides et al., 2023). However, no maternal variables emerged as predictors of CU traits in women (Kyranides et al., 2023).

One of the studies identified discrete early risk factors, namely parental criminality, authoritarian parenting, inadequate supervision, interparental conflict, and premature parenthood, as significant predictors of female psychopathy (Farrington & Bergstrøm, 2023). These factors demonstrated differential associations with Factor 1 (psychopathic personality; F1-PP) and Factor 2 (psychopathic behaviour; F2-PB). The adversities identified showed substantial overlap with conditions linked to the development of insecure or disorganized attachment, suggesting attachment-related mechanisms may mediate the relationship between environmental risk exposure and psychopathic trait emergence (Farrington & Bergstrøm, 2023).

The only qualitative study in this systematic review (Arrigo & Griffin, 2004) proposed that exposure to inconsistent parenting and abandonment experiences may facilitate the development of avoidant attachment orientations characterized by emotional detachment, hostility, social isolation, behavioural impulsivity, and diminished interpersonal sensitivity, all factors that significantly impair the capacity for secure attachment formation and meaningful relational engagement. The case study of Aileen Wuornos (Arrigo & Griffin, 2004) illuminates how inconsistent early attachment experiences disrupt emotional regulation development and adaptive coping mechanisms. Inconsistent internal working models established during formative periods manifest in adulthood as emotional expression deficits, pervasive mistrust, and maladaptive coping strategies, including substance abuse, violence, and criminal behaviour (Arrigo & Griffin, 2004). The absence of internalized representations of secure attachment predisposed Wuornos to victim devaluation, facilitating calculated, remorseless violent acts. Her fundamental attachment impairment enabled the perpetration of criminal behaviours without empathic inhibition or remorse (Arrigo & Griffin, 2004), illustrating the profound implications of early attachment disruption for antisocial behaviour development.

Although only one study was conducted in a prison setting, some differences emerged between prison and community contexts in studies examining early life experiences and attachment-related factors. The prison-based study (Eisenbarth et al., 2018) highlighted maternal influences, showing that abusive maternal practices were stronger predictors of psychopathy, particularly the lifestyle facet, than paternal ones. In contrast, in community samples, Kyranides et al. (2023) found that paternal factors, specifically the sense of responsibility towards the father, were positive predictors of CU traits in women, whereas no maternal variables showed significant effects. Other community-based studies (e.g., Bloxsom et al., 2021; Farrington & Bergstrøm, 2023) reinforced the role of broader family risks such as parental criminality, poor supervision, and interparental conflict.

## 18. Discussion

This systematic review synthesizes current evidence on the relationship between attachment and related factors, early life experiences, and psychopathic traits in women, highlighting several key themes: an association between insecure attachment patterns—particularly avoidant and anxious styles—and primary psychopathic traits (e.g., interpersonal manipulation, emotional detachment, lack of empathy); the influence of early caregiving relationships, with maternal attachment being especially salient; and the role of maladaptive schemas mediating the relationship between childhood adversity and psychopathy development in women (see Table 4). These findings suggest a multifactorial developmental pathway to psychopathy in women, grounded in early attachment dynamics and further shaped by environmental and relational experiences.

The research suggests an association between anxious and avoidant attachment styles and psychopathic traits, although more empirical evidence is needed to confirm this (Bloxsom et al., 2021; Eisenbarth et al., 2018). These results seem to find more consistency in the primary characteristics of psychopathy in women, where both attachment styles seem to be correlated with the development of the primary characteristics of psychopathy, namely interpersonal manipulation, lack of empathy, superficial affection, and selfishness (Mooney et al., 2019). However, it is important to note that most of the included studies are cross-sectional and conducted in community settings, which limits the ability to draw firm inferences or identify developmental pathways. These limitations highlight the need for further research employing longitudinal designs and involving diverse samples, including forensic and clinical populations.

Additionally, the positive correlations between anxious and avoidant attachment styles and psychopathy (Bloxsom et al., 2021) shed light on the complex interplay between attachment dynamics and the development of psychopathic traits. This finding is consistent with existing literature, suggesting that anxious and avoidant attachment styles may be associated with distinct coping strategies and patterns of social interaction (Mikulincer & Shaver, 2012). These correlations underscore the nuanced relationship between attachment styles and psychopathology, pointing to the importance of considering individual attachment patterns in understanding the development of psychopathic characteristics.

The association between avoidant attachment and psychopathic traits aligns with Bowlby (1969) and Ainsworth et al.’s (1978) theories, suggesting that early inconsistent or negligent care experiences can lead to the formation of an insecure working model characterized by distrust in others and a negative self-view. Additionally, one study reinforces the link between avoidant attachment and CU traits in psychopathy (Kyranides et al., 2023), highlighting the significance of avoidant attachment in understanding emotional detachment and interpersonal difficulties typical of psychopathy. According to attachment theory, children with avoidant attachment tendencies develop coping strategies that minimize or deny the importance of emotions (Mikulincer & Shaver, 2019), potentially leading to suppressed empathic emotions and emotional detachment, traits often observed in individuals with psychopathic tendencies (Decety et al., 2013). This perspective is supported by research such as Lyons-Ruth and Jacobvitz (2016), indicating that the lack of caregiver sensitivity and responsiveness during childhood correlates with later difficulties in emotional regulation and the establishment of healthy relationships.

Additionally, the differential impact of maternal and paternal parenting styles on psychopathic traits in women (Eisenbarth et al., 2018) suggests that maternal attachment plays a more significant role in shaping behavioural outcomes of psychopathy. The attachment theory suggests that the initial bond between mother and child plays a key role in the child’s emotional and behavioural development (Bowlby, 1969), so attachment experiences with the mother may have a longer-lasting impact on psychopathic traits in women than attachment experiences with their father. In addition, some results found in the literature show that mothers often play a central role in emotional socialization and empathy development in children (B. L. Blair et al., 2014), and, therefore, mother–child interactions play a more significant role in internalization of social norms and emotional regulation, thus affecting the expression of psychopathic traits in women. Nevertheless, the predominance of studies based on community samples raises concerns about the generalizability of these associations to forensic or clinical populations, where relational and developmental dynamics may differ significantly.

This review emphasizes the significance of paternal involvement as a protective factor against the development of psychopathy in women (Arrigo & Griffin, 2004). This finding underscores the importance of early caregiving experiences in influencing psychosocial outcomes, aligning with attachment theory principles proposed by Bowlby (1969). It also supports research indicating the father’s role in teaching emotional regulation skills and establishing appropriate boundaries, which improve emotional, behavioural, and cognitive outcomes in children (Sarkadi et al., 2008). The review highlights the significance of emotional regulation, often impaired in psychopathy, leading to maladaptive strategies and influencing psychopathy traits among women (Kyranides & Neofytou, 2021). Analysing Aileen’s case highlights her difficulties in forming attachments, which may reflect early maladaptive attachment patterns that influence emotional regulation (Arrigo & Griffin, 2004). These patterns can lead to a lack of empathy towards victims and aggressive interactions, as observed in psychopathic behaviours. This relationship between maladaptive emotional regulation and psychopathy underscores the necessity for targeted interventions focusing on improving emotion regulation skills among individuals at risk for developing psychopathy. Research supports these findings and suggests that gender-specific differences in emotional regulation strategies should be considered in clinical interventions (Freitas et al., 2024; Matud, 2004). However, it is important to note that while the interpretations drawn from psychoanalytic perspectives, such as those by Arrigo and Griffin (2004), provide valuable insights, they represent only one of many possible frameworks for understanding these phenomena. In addition, the negative correlation between avoidant attachment and cognitive and affective empathy reinforces the role of attachment patterns in shaping empathic abilities (Bloxsom et al., 2021). Individuals with avoidant attachment inhibit or block the activation of the attachment system to maintain suppressed attachment needs and tendencies, leading to inhibition of emotional experiences (Mikulincer & Shaver, 2019). This lack of emotional sensitivity can result in a lower ability to feel cognitive and affective empathy with others, making it difficult to infer thoughts, feelings, and intentions of others, often compromised in individuals with psychopathic traits in response to deficits in emotional processing (R. J. Blair, 2007; Pinheiro et al., 2023).

The exploration of early life experiences provides valuable insights into the developmental pathways leading to psychopathy in women (Doroszczyk & Talarowska, 2023; Farrington & Bergstrøm, 2023). These studies underscore the long-term impact of early experiences on psychosocial development. Simard et al. (2011) found that young adults with insecure-ambivalent child attachment or insecure-preoccupied adult attachment styles showed more signs of early maladaptive schemas compared to their secure peers. This suggests that unmet childhood needs for secure attachment may lead to a variety of early maladaptive schemas, consequently affecting the severity of psychopathy in women (Doroszczyk & Talarowska, 2023; Simard et al., 2011). Additionally, the identification of specific childhood risk factors, such as parental conflict and authoritarian parenting (Farrington & Bergstrøm, 2023), highlights the need for early intervention and support systems to mitigate the impact of adverse childhood experiences on psychosocial outcomes.

Despite the aforementioned, the methodological diversity of attachment measures used across the included studies significantly constrains the robustness of the conclusions. Over recent decades, a wide range of approaches has been developed and validated to assess attachment, including narrative interviews, self-report questionnaires, script-based tasks, and priming methods (Waters et al., 2000). This multiplicity of strategies raises a fundamental question: is there a common core that defines attachment relationships and is consistently captured by these measures? Current evidence suggests otherwise. As highlighted by Crowell (2021), widely used tools such as the Adult Attachment Interview and the Experiences in Close Relationships do not predict outcomes in the same way, despite both being related to adult relational functioning. This inconsistency is also reflected in the literature on the stability and change of attachment over time, where different instruments yield divergent estimates (Thompson et al., 2022). Furthermore, there is increasing recognition that changes in attachment orientations are not merely exceptions or methodological errors but are an inherent part of relational development—referred to as “lawful discontinuity” (Fraley & Dugan, 2021). Significant relational changes, such as entering new social roles, exposure to chronic stress, or the influence of romantic partners, may trigger legitimate shifts in secure attachment during adulthood (Thompson et al., 2022). This dynamic conceptualisation of attachment becomes particularly relevant when considering clinical samples and high-risk contexts, such as women with disruptive relational patterns or a history of trauma. Thus, the apparent inconsistency in findings across the reviewed studies may reflect not only methodological differences but also legitimate and expected changes in attachment experiences over the course of development. Finally, although dimensional measures of attachment offer psychometric advantages—such as consistency across life stages and relationship types—authors like Steele and Steele (2021) argue that these approaches may not adequately capture the relational quality involved, particularly in contexts of disorganised attachment or unresolved trauma. Therefore, the choice of assessment tool should be carefully aligned with the aims and characteristics of the population under study. Methodological plurality will, therefore, continue to represent a challenge to drawing generalisable conclusions about attachment, underscoring the importance of a cautious and context-sensitive interpretation of results. A similar concern applies to the assessment of psychopathy. The studies included in this review employed a range of instruments—spanning from clinically administered tools such as the PCL-R to self-report measures like the LSRP—each rooted in distinct theoretical frameworks and emphasizing different facets of the construct (Hare, 2003). This variability reflects ongoing conceptual debates in the field (e.g., categorical vs. dimensional models; emphasis on interpersonal/affective vs. behavioural dimensions) and contributes to the heterogeneity observed across findings (Skeem et al., 2003). Taken together, these limitations highlight the need for caution in interpreting the findings of this review. The heterogeneity of samples and measurement tools, along with the reliance on cross-sectional, community-based studies, precludes causal interpretations and limits the generalizability of the results.

## 19. Conclusions: Limitations and Future Research

Although the findings are important, this systematic review identified several limitations. First, most of the included studies employed cross-sectional and retrospective designs (e.g., Bloxsom et al., 2021; Eisenbarth et al., 2018; Kyranides & Neofytou, 2021), which hinder the ability to infer causality and to trace developmental trajectories over time. Future research should prioritize longitudinal designs to clarify the developmental pathways linking attachment and psychopathic traits. While attachment theory (Bowlby, 1969, 1988) posits that early relational experiences shape long-term emotional and personality development, the notion that attachment patterns remain stable across the lifespan has been increasingly contested. Findings from longitudinal studies have been mixed—some indicating moderate continuity from childhood to adulthood (e.g., Hamilton, 2000; Waters et al., 2000), while others report little to no stability (e.g., Lewis et al., 2000). Second, the predominance of community samples limits the applicability of current findings to forensic and clinical contexts. To enhance generalizability and relevance to high-risk populations, future studies should include clinical and forensic samples. This would strengthen the evidence base for informing prevention efforts, risk assessment, and intervention planning. Third, the exclusive reliance on self-report instruments, which are often vulnerable to social desirability bias (e.g., Bloxsom et al., 2021; Walsh et al., 2019), and the limited sample sizes in most studies (Eisenbarth et al., 2018; Walsh et al., 2019) further restrict the external validity. The adoption of multiple and complementary methods, such as semi-structured interviews, behavioural tasks, and self-reported measures (by parents, peers, or intimate partners), could offer a more comprehensive and objective view of interpersonal dynamics and psychopathic manifestations (Miller et al., 2011). This methodological triangulation is especially important in clinical and forensic samples, where the exclusive use of self-reported measures may be particularly limited. Fourth, the use of diverse assessment tools across studies to evaluate psychopathic traits, attachment styles, and related variables (e.g., parenting care) introduces inconsistencies that challenge comparability and synthesis of results. These inconsistencies are compounded by methodological and conceptual differences between developmental and adult attachment research traditions, including the use of distinct measures and theoretical frameworks (Raby et al., 2015). In line with this, a narrative synthesis was chosen over a meta-analysis due to the considerable heterogeneity among the included studies. Specifically, there were substantial differences in the conceptualization and operationalization of psychopathy (e.g., use of different instruments, cut-off points, and theoretical frameworks), as well as in the definition and measurement of attachment styles (e.g., categorical vs. dimensional approaches). In addition, variations in sample characteristics (e.g., forensic vs. community samples, different age ranges), methodological quality, and study design further limited the feasibility of a statistically meaningful meta-analysis. A related concern is the issue of measurement invariance, as many attachment and psychopathy instruments were originally developed and validated in male samples. This raises important questions about their comparability across genders and between different assessment tools, potentially impacting the consistency of findings and the validity of conclusions (Klein Haneveld et al., 2022). A narrative synthesis was therefore deemed the most appropriate strategy to provide a coherent overview of the existing evidence, while also allowing for a more nuanced interpretation of patterns and inconsistencies across studies. Moreover, the overall methodological quality of the included studies was frequently rated as moderate, as assessed using the Mixed Methods Appraisal Tool (MMAT; Q. Hong et al., 2018), which warrants a cautious interpretation of the findings presented in this review. Moderate methodological quality may reflect critical limitations, such as inadequate control for confounding variables, the use of measurement tools with limited validity, or insufficient transparency in the reporting of methodological procedures. These shortcomings undermine not only the robustness of the conclusions but also their reliability and replicability across different contexts (Higgins et al., 2017). Therefore, future research should adopt more rigorous methodological standards and clearly defined quality criteria to strengthen the empirical evidence in this field. In addition, unmeasured confounding variables—such as trauma history and psychiatric comorbidities—may have influenced the observed associations. Childhood adversities, including abuse and neglect, are strongly associated with both insecure attachment and psychopathic traits, particularly secondary psychopathy (Di Giacomo et al., 2021). Similarly, comorbid conditions such as PTSD, substance use disorders, and mood disorders—which are highly prevalent among women with psychopathic traits—can affect both attachment patterns and emotion regulation. These overlapping influences complicate the interpretation of findings (Killeen & Brewerton, 2023). To clarify the unique contribution of attachment to psychopathy in women, future research should systematically control for these confounders. Fourth, while psychoanalytic interpretations (e.g., Arrigo & Griffin, 2004) provide valuable insights into relational dynamics and psychopathy, they represent only one of many theoretical approaches. Future research should adopt diverse methodological frameworks, incorporating empirical studies grounded in psychological, genetic, and neurobiological perspectives to advance a more integrative understanding of female psychopathy. Fifth, a notable geographic bias was observed, with most studies conducted in the UK and US. This limited scope highlights the absence of research in other cultural contexts, which is essential for capturing the full complexity of how psychopathic traits manifest in women worldwide. The lack of global representation likely reflects a broader neglect of female-specific pathways in psychopathy research, particularly within offending populations. Addressing this gap requires a concerted effort from the scientific community to promote inclusive and well-funded research agendas that explore the nuances of female psychopathy from a global perspective. Equally relevant is the lack of participant diversity in many studies, which often fail to consider variables such as socioeconomic status, ethnicity, sexual orientation, gender identity, religion, age, functional capacity, or geographic context. This lack of inclusion compromises the generalizability of the results. Future research should adopt an intersectional approach that recognizes the influence of multiple social determinants on the expression of attachment and psychopathic traits in women. Despite these limitations, this systematic review underscores the value of integrating attachment theory into the understanding of female psychopathy, with substantial implications for research, clinical practice, and policy (see Table 5). From a preventive standpoint, early intervention programs that promote secure attachment—particularly in high-risk contexts marked by trauma, neglect, or instability—may reduce the likelihood of developing maladaptive traits associated with psychopathy (McDonald et al., 2011). Attachment-informed initiatives, such as parenting programs and trauma-sensitive school practices, are crucial not only in childhood but also during adolescence, a critical period for reshaping internal working models (Dozier et al., 2008).

Women with psychopathy and insecure attachment face unique challenges in treatment, which differ from the difficulties faced by men (Wynn et al., 2012). The relational and developmental experiences of women shape the expression of psychopathy, complicating therapeutic outcomes. Women with insecure attachment tend to struggle with trust and emotional regulation, making treatment engagement more challenging (Velotti et al., 2015). Existing interventions often fail to address these attachment-related difficulties, highlighting the need for gender-sensitive approaches that consider the emotional and relational complexities specific to women. In practical terms, avoidant attachment in women may serve as a clinical risk marker for elevated CU and affective traits—characterized by reduced empathy and interpersonal detachment—which are critical considerations in forensic risk assessment. In contrast, anxious attachment may indicate vulnerability to emotion dysregulation and secondary psychopathy, suggesting the need for distinct strategies in risk management and intervention planning.

With regard to public policy, the findings underscore the urgent need to incorporate attachment-informed models into the planning and delivery of psychosocial support services, both within institutional settings and in community reintegration programs. Interventions should be designed not only to address behavioural outcomes but also to promote relational security and emotional resilience among women with histories of early adversity. This approach aligns with contemporary calls for gender-responsive rehabilitation models that acknowledge the distinct developmental and psychosocial trajectories of female offenders (Covington, 2007). Accordingly, tailored interventions should be grounded in attachment theory and trauma-informed care, with a focus on enhancing secure attachment, repairing early relational disruptions, and strengthening emotion regulation capacities. In particular, women exhibiting elevated CU or affective traits may benefit from attachment-based interventions such as Mentalization-Based Treatment (MBT) and Attachment-Based Family Therapy (ABFT), which specifically target empathic engagement and the development of emotional regulation skills. These approaches have the potential to strengthen the therapeutic alliance, reduce interpersonal coldness, and enhance treatment adherence. Evidence-based approaches such as Mentalization-Based Treatment (MBT) and Attachment-Based Family Therapy (ABFT) have shown promise in this regard (Fonagy & Allison, 2014; Diamond et al., 2016), offering valuable frameworks for promoting psychological well-being and reducing recidivism among justice-involved women. Integrating attachment-based approaches into mental health and criminal justice systems is therefore essential for providing appropriate and effective care to women presenting with psychopathic traits. Current public policies should prioritize the systematic inclusion of attachment theory in the development of treatment protocols specifically tailored to the needs of female offenders.

From a clinical perspective, we argue that early screening for dysfunctional attachment configurations—particularly in populations with a known history of trauma or adversity—can guide the development of specific attachment-based therapeutic interventions. These interventions could promote better emotion regulation, interpersonal functioning, and reduce the expression of externalizing behaviours often associated with secondary psychopathy (Frick et al., 2014). These perspectives are especially relevant in forensic and correctional settings, where the application of trauma-informed and gender-sensitive care remains limited, despite empirical evidence supporting its effectiveness (Van Voorhis et al., 2010).

In conclusion, this systematic review underscores the importance of incorporating attachment theory into the study of psychopathy in women. The findings suggest that attachment styles and other related factors may play an important role in shaping the expression of psychopathic traits, potentially influencing how such traits manifest and develop over time. The insights provided are valuable for researchers, clinicians, and policymakers. Future research should prioritise greater theoretical and methodological integration between developmental and adult attachment models, as existing frameworks often rely on non-equivalent constructs and assessment tools, hindering longitudinal continuity (Thompson et al., 2022). A promising direction involves the centralisation of internal working models as a core construct for understanding attachment across the lifespan. Advancing the direct assessment and conceptual refinement of internal working models may increase both theoretical coherence and clinical relevance (Schuengel et al., 2021).

Attachment research faces the challenge of aligning scientific concepts, such as “sensitivity” and “attachment,” with their meanings in the public sphere to enhance societal impact (Duschinsky et al., 2021). To achieve this, it is essential to prioritize dissemination and knowledge translation, focusing on concrete and socially relevant issues, such as equitable access to essential early childhood care (Britto et al., 2017). Adopting this approach, along with conceptual clarification and addressing “Goldilocks problems”—those complex enough for scientific advancement yet amenable to practical solutions—can increase the relevance and influence of attachment research on policy and practice. Future efforts should focus on the development of targeted, gender-sensitive interventions grounded in attachment theory to better address the needs of women with psychopathic traits and to improve treatment outcomes and public safety.

## Figures and Tables

**Table 1:** Summary of the studies’ objectives, measures, and main outcomes.

Study	Variable(s)| Objective(s)	Measures		Main Outcomes	Key Themes
		Attachment	Psychopathy		
Arrigo and Griffin (2004)	Examine attachment theory, psychopathy and predatory homicide relationship	Books, taped interviews, television programs, and newspaper articles, chronicling the life events of Aileen Wuornos		Aillen’s Childhood: Experienced inconsistent care from grandmother and grandfather. Experienced sexual abuse from her grandfather. Separation from mother; lack of emotional support. Developed avoidant attachment style. Fragmented sibling attachments; lacked healthy models of love and affection. Aillen’s Adolescence: Developed self-hatred and animosity towards others. Aillen’s Adult Life: Displayed avoidant and hostile behaviour: detachment and social withdrawal. Engaged in criminal activities with low empathy (psychopathic traits). Distrustful and emotionally suppressed. Coping mechanisms included drug abuse, prostitution, violence, and an unstable lifestyle. Victim and Perpetrator: Shifted from being a victim in childhood to a perpetrator in adulthood. Insecure attachment facilitated devaluation of victims and cold, remorseless psychopathic behaviour.	Early Life Experiences, Attachment Styles, and Psychopathy
Bloxsom et al. (2021)	Investigate the interrelationships of the Dark Triad and its unique trait associations with parental and intimate adult (best friend/partner) attachment difficulties, along with the mediating role of empathic deficits.	Experiences in Close Relationships-Revised (ECR-R): anxiety attachment and avoidant attachment	Dark Triad of Personality—Short Version (Machiavellianism, Narcissism, and Psychopathy)	Anxious and avoidant parental attachment positively correlated with psychopathy; avoidant parental attachment also correlated with Machiavellianism. Machiavellianism and psychopathy positively correlated with anxious and avoidant adult attachment to best friends/partners. Cognitive and affective empathy negatively correlated with avoidant attachment and positively with anxious attachment in best friend relationships. Insecure parental attachment predicts psychopathy and Machiavellianism, though weakly. Psychopathy is directly and indirectly linked to insecure adult intimate attachments. Women with avoidant parental attachment are more likely to exhibit Machiavellian and psychopathic traits, influencing adult intimate relationships.	Attachment Styles and Psychopathy
Doroszczyk and Talarowska (2023)	Examine the relationship between Young’s early maladaptive schemas and psychopathic personality traits in a non-clinical population.	Young’s Schema Questionnaire (YSQ-S3-PL)	Psychopathic Personality Traits Scale—Revised (PPTS-R) (Affective Responsiveness, Cognitive Responsiveness, Interpersonal Manipulation, and Egocentricity) Triarchic Psychopathy Measure (TriPM) (Boldness, Meanness, and Disinhibition)	Correlations between psychopathy severity and early maladaptive schemas: Psychopathy severity (TriPM) positively correlates with multiple maladaptive schemas, notably emotional deprivation, mistrust/abuse, entitlement/grandiosity, insufficient self-control, and approval seeking. The most strongly associated domain with psychopathy was impaired limits. Correlations between psychopathic traits and early maladaptive schemas: In total, 13 of 18 schemas from PPTS-R positively correlate with psychopathic traits, with entitlement/grandiosity strongest, followed by the rejection domain.	Early Life Experiences, Attachment-related Factors, and Psychopathy
Eisenbarth et al. (2018)	Investigating the factor structure and construct validity of the PCL-R in a Hispanic female sample	Measure of Parenting Style (MOPS)	Psychopathy Checklist Revised (PCL-R) (Interpersonal, Affective, Lifestyle, and Antisocial factors) NEO-PI Five-Factor Inventory (FFI) (Openness, Conscientiousness, Extraversion, Agreeableness, Neuroticism)	Indifference and abusive parenting styles are positively associated with higher psychopathic traits. Abusive maternal style is significantly linked to the lifestyle facet of psychopathy. Psychopathic traits correlate strongly with both maternal and paternal parenting styles. Maternal styles have a stronger direct effect on psychopathy than paternal styles. The influence of parenting styles on psychopathy is partly mediated by the Five Factor Model (FFM).	Early Life Experiences, Attachment-related Factors, and Psychopathy
Farrington and Bergstrøm (2023)	Identify the main risk factors for psychopathy in men and women. Compare the differences in risk factors for psychopathy between men and women. Determine which risk factors are independently predictive of psychopathy in men and women.	Parental Attitude Schedules developed: retrospective questions about their childhood	Psychopathy Checklist: Screening Version (PCL:SV) (F1-PP Factor 1, psychopathic personality; F2-PB Factor 2, psychopathic behaviour)	Relationship between psychopathy and convictions: Psychopathic traits are not associated with convictions in women. Predictive factors for psychopathy: Young mothers, convicted mothers, parental conflict, and authoritarian fathers were predictive of psychopathy in women. Risk factors: Convicted parents, poor supervision, and lack of attention are prevalent risk factors among both men and women. Relationships between risk factors and psychopathy in women: Young mothers, poor supervision, risk-taking behaviour, and early school leaving are strong risk factors associated with psychopathy. Risk factors independently predictive: For women, risk-taking, parental conflict, low take-home pay, and poor supervision are independent predictors of psychopathy. Other predictors for continuous psychopathy include factors like young mother, separation, risk-taking, parental conflict, early school leaving, lack of attention, authoritarian parenting, and physical punishment.	Early Life Experiences, Attachment-related Factors, and Psychopathy
Kyranides and Neofytou (2021)	Explore the relationship between adult insecure attachment dimensions and CERQ with both primary and secondary traits	Relationship Scale Questionnaire (RSQ): measure adult attachment in terms of general orientations to close and interpersonal relationships (anxiety and avoidance attachment)	Levenson Self-Report Psychopathy Scale (LSRP) (Primary and Secondary Psychopathy)	Gender differences in psychopathic traits, attachment, and cognitive emotion regulation strategies (CERS): Men show higher primary and secondary psychopathic traits; women report more rumination (maladaptive CERS). Secondary psychopathic traits positively correlate with anxious and avoidant attachment; primary traits show no attachment correlation. Primary psychopathic traits correlate positively with maladaptive CERS (catastrophizing) and adaptive CERS (positive reappraisal). Secondary psychopathic traits correlate positively with maladaptive CERS and negatively with adaptive CERS. Avoidant attachment predicts primary psychopathic traits; anxious attachment predicts secondary traits. “Blaming others” predicts primary psychopathy positively; “putting things in perspective” predicts it negatively. “Catastrophizing” is a positive predictor for secondary psychopathy; self-blame and rumination are not significant predictors. Gender and age contribute to predicting psychopathic traits, with men more likely to exhibit higher levels.	Attachment Styles and Psychopathy
Kyranides et al. (2023)	To explore how anxious and avoidant attachment dimensions, maternal and paternal factors predict CU traits in women and men separately	The Relationship Scales Questionnaire (RSQ) Parent Adult-Child Relationship Questionnaire (PACQ)	Inventory of Callous- Unemotional Traits (ICU) (Callousness, Unemotional, and Uncaring)	Gender differences in CU traits: Women report lower levels of CU traits compared to men. Role of attachment: Attachment dimensions (avoidance and anxiety) explain the largest variance of CU traits in both men and women. Avoidant attachment is a significant positive predictor of CU traits in both genders. CU traits are positively correlated with anxious attachment in both genders. Age and anxious attachment are significant negative predictors of CU traits in both men and women. Women report higher levels of attachment-related anxiety than men. Maternal and paternal factors: CU traits are positively correlated with avoidance in both genders. The factor assessing responsibility towards the father positively predicts CU traits only in women. Both mother and father responsibility and father regard variables are significantly higher in men compared to women.	Attachment Styles and Psychopathy
Walsh et al. (2019)	Examine whether the four factors of psychopathy were differentially associated with attachment domains and whether there were gender differences in the associations between psychopathic traits and attachment problems	Experiences in Close Relationships (ECR)	Self-Report Psychopathy-Short Form (SRP-SF) (Interpersonal, Affective, Lifestyle, and Antisocial factors)	Attachment and psychopathic traits in women: The affective factor positively affects avoidant attachment, while the antisocial factor negatively affects anxious attachment. Attachment and psychopathic traits in men: The affective factor has a similar positive effect on avoidant attachment, while the lifestyle factor positively affects anxious attachment. Association between affective psychopathic traits and attachment: Affective psychopathic traits show moderately strong associations with avoidant attachment across genders. Psychopathic traits and attachment style: Psychopathic traits, especially affective traits, are linked to avoidant attachment style. Differential effects of psychopathic characteristics on attachment: The lifestyle and antisocial characteristics of psychopathy are differentially linked to anxious attachment for men and women. Impact on seeking comfort and closeness: Women with psychopathic traits, particularly those engaged in overtly antisocial and criminal acts, appear less likely to seek comfort and closeness with others.	Attachment Styles and Psychopathy

Note. QA = Quality Assessment.

**Table 2:** Study design and characteristics of the sample.

Study	Design Type	Location and Setting	Sample Characteristics		
			Sex|Age (M/SD)	Size (n)	Ethnicity/Race
Arrigo and Griffin (2004)	Qualitative Case study	USA; N/A	woman Aileen Wuornos	1 woman	N/A
Bloxsom et al. (2021)	Quantitative non-randomized study	United Kingdom; Community	Women (*M* = 26.65, *SD* = 11.65) Range 18 to 71 years	262 women	White British (42.4%), White Other (31.7%), Asian (8.4%), Hispanic or Latino (5.0%), Black or African American (2.7%), Native American (1.1%), Other (5%) and ‘Prefer not to say’ (3.8%)
Doroszczyk and Talarowska (2023)	Quantitative non-randomized study	N/A; Community	women and men Range 18 to 45 years	150 (129 women)	N/A
Eisenbarth et al. (2018)	Quantitative non-randomized study	USA; Prison	women offenders (*M* = 34.23; *SD* = 7.06); Range 21 to 54 years	155 women offenders	Hispanic
Farrington and Bergstrøm (2023)	Descriptive—Quantitative and Qualitative	United Kingdom; Community	women and men *M* = 25.4 (women)	551 (260 women)	N/A
Kyranides and Neofytou (2021)	Quantitative non-randomized study	United Kingdom; Community	women and men (*M* = 35.11, *SD* = 13.49) Range 18 to 70 years	338 (231 women)	N/A
Kyranides et al. (2023)	Quantitative non-randomized study	United Kingdom; Community	women and men (*M* = 30.96, *SD* = 11.66) Range 18 to 72 years	1149 (752 women)	N/A
Walsh et al. (2019)	Quantitative non-randomized study	USA; Community	women and men (*M* = 20.52, *SD* = 3.98) for women	590 (206 women)	European Americans (64.6%), African Americans (14.9%), Asian Americans (7.6%), Latino/Hispanic (10%)

Note. *M* = Mean; *SD* = Standard Deviation.

**Table 3:** Quality assessment of included studies.

Authors	Screening Questions		Quantitative Descriptive					Qualitative					Mixed Methods					QA
	S1	S2	Q1	Q2	Q3	Q4	Q5	Q1	Q2	Q3	Q4	Q5	Q1	Q2	Q3	Q4	Q5	
Arrigo and Griffin (2004)	Yes	Yes	-	-	-	-	-	Yes	No	Yes	Yes	Yes	-	-	-	-	-	4
Bloxsom et al. (2021)	Yes	Yes	Yes	Yes	Yes	Yes	Yes	-	-	-	-	-	-	-	-	-	-	5
Doroszczyk and Talarowska (2023)	Yes	Yes	Yes	Yes	Yes	Yes	No	-	-	-	-	-	-	-	-	-	-	4
Eisenbarth et al. (2018)	Yes	Yes	Yes	No	Yes	Yes	Yes	-	-	-	-	-	-	-	-	-	-	4
Farrington and Bergstrøm (2023)	Yes	Yes	-	-	-	-	-	-	-	-	-	-	Yes	Yes	Yes	Yes	No	4
Kyranides and Neofytou (2021)	Yes	Yes	Yes	Yes	Yes	Yes	Yes	-	-	-	-	-	-	-	-	-	-	5
Kyranides et al. (2023)	Yes	Yes	No	Yes	Yes	Yes	No	-	-	-	-	-	-	-	-	-	-	3
Walsh et al. (2019)	Yes	Yes	Yes	Yes	Yes	Yes	Yes	-	-	-	-	-	-	-	-	-	-	5

Note. QA = Quality assessment; screening questions: S1. Are there clear research questions? S2. Do the collected data allow to address the research questions? Quantitative descriptive study: Q1. Is the sampling strategy relevant to address the research question? Q2. Is the sample representative of the target population? Q3. Are the measurements appropriate? Q4. Is the risk of nonresponse bias low? Q5. Is the statistical analysis appropriate to answer the research question? Qualitative study: Q1. Is the qualitative approach appropriate to answer the research question? Q2. Are the qualitative data collection methods adequate to address the research question? Q3. Are the findings adequately derived from the data? Q4. Is the interpretation of results sufficiently substantiated by data? Q5. Is there coherence between qualitative data sources, collection, analysis, and interpretation? Mixed methods study: Q1. Is there an adequate rationale for using a mixed methods design to address the research question? Q2. Are the different components of the study effectively integrated to answer the research question? Q3. Are the outputs of the integration of qualitative and quantitative components adequately interpreted? Q4. Are divergences and inconsistencies between quantitative and qualitative results adequately addressed? Q5. Do the different components of the study adhere to the quality criteria of each tradition of the methods involved?

**Table 4:** Key findings of the systematic review.

Attachment Styles and Psychopathy: Avoidant attachment predicts callous-unemotional traits, while anxious attachment functions as a negative predictor of psychopathy in women. Empathy (both cognitive and affective) negatively correlates with avoidant attachment, with relationship-specific variations in these associations. Insecure attachment patterns contribute to emotional detachment and interpersonal dysfunction characteristic of psychopathic presentation. Early Life Experiences, Attachment-related Factors, and Psychopathy: Parental influences shape psychopathy development, with negative practices (abandonment, indifference, abuse) increasing risk and positive involvement acting as a protective factor. Maternal negative parenting demonstrates stronger direct effects on psychopathic traits compared to paternal influences, which operate through more indirect pathways. Early caregiving disruptions (family criminality, inconsistent parenting, parental conflicts, early parenthood) significantly predict psychopathic traits in women. Maladaptive cognitive schemas (particularly entitlement/grandiosity and insufficient self-control) mediate the relationship between adverse childhood experiences and psychopathy development.

**Table 5:** Implications for research, practice, and policy.

Implications for Research	Implications for Practices and Policy
Expand the cultural and sociodemographic diversity of samples to understand how intersectional factors (gender, class, ethnicity, sexual orientation, etc.) modulate the relationship between attachment and psychopathy. Develop longitudinal studies to examine the evolution of attachment configurations and psychopathic traits over time, clarifying the directionality of the association. Investigate integrative models that connect dimensions of attachment (e.g., Internal Working Models) with neurobiological, emotional, and social factors in the expression of female psychopathy. Promote greater theoretical and methodological integration between research on childhood and adult attachment, using compatible constructs and instruments across the lifespan. Use mixed methods and methodological triangulation (self-report, interviews, behavioural measures) to reduce social desirability bias and enhance data validity.	Interventions should emphasise the promotion of secure attachment and positive parenting practices to mitigate the risk of psychopathy in women. Sex differences in attachment styles and psychopathic traits emphasize the need for personalized interventions that meet the unique needs of women with psychopathic traits. Develop attachment-based interventions, such as MBT (Mentalization-Based Treatment) and ABFT (Attachment-Based Family Therapy), tailored to the emotional and relational profiles of women. Early intervention programs should be implemented to address risk factors associated with psychopathy, such as parental conflict and negative parenting styles. Adapt therapeutic programmes to be gender-sensitive, addressing the specific difficulties of women with insecure attachment, such as mistrust, fear of rejection, or emotional dysregulation. Apply attachment-based approaches in community settings, such as schools, primary healthcare, and family support centres, rather than limiting their use to clinical or correctional environments. Policies aimed at improving access to mental health services and support for individuals with psychopathic traits, especially women, could contribute to better outcomes and reduced recidivism rates. Promote positive parenting programmes and early family interventions in contexts of adversity to help prevent the intergenerational transmission of psychopathic traits and insecure attachment. Encourage policies that ensure equitable access to early childhood care, such as emotionally attuned daycare services, particularly in socioeconomically vulnerable areas. Support emotional literacy and parenting campaigns that make key attachment concepts accessible to the general population Integrate attachment dimensions (e.g., avoidant and anxious styles) into forensic risk assessment protocols to better identify women with higher CU/affective traits and guide individualized treatment planning. Women with elevated CU traits may benefit from attachment-based interventions (e.g., MBT, ABFT) that target empathic engagement, emotion regulation, and treatment adherence.

## Data Availability

The data that support the findings of this study are available from the corresponding author, MP, upon reasonable request.
